# Metabolic alterations mediated by STAT3 promotes drug persistence in CML

**DOI:** 10.1038/s41375-021-01315-0

**Published:** 2021-06-12

**Authors:** Sweta B. Patel, Travis Nemkov, Davide Stefanoni, Gloria A. Benavides, Mahmoud A. Bassal, Brittany L. Crown, Victoria R. Matkins, Virginia Camacho, Valeriya Kuznetsova, Ashley T. Hoang, Danielle E. Tenen, Samuel L. Wolock, Jihye Park, Li Ying, Zongliang Yue, Jake Y. Chen, Henry Yang, Daniel G. Tenen, Paul Brent Ferrell, Rui Lu, Victor Darley-Usmar, Angelo D’Alessandro, Ravi Bhatia, Robert S. Welner

**Affiliations:** 1 Department of Medicine, Division of Hematology/Oncology, O’Neal Comprehensive Cancer Center, University of Alabama at Birmingham, Birmingham, AL USA; 2 Department of Biochemistry and Molecular Genetics, School of Medicine, University of Colorado Anschutz Medical Campus, Aurora, CO USA; 3 Department of Pathology, Center for Free Radical Biology, University of Alabama at Birmingham, Birmingham, AL USA; 4 Department of Systems Biology, Harvard Medical School, Boston, MA USA; 5 Cancer Institute of Singapore, National University of Singapore, Singapore, Singapore; 6 Division of Endocrinology, Beth Israel Deaconess Medical Center, Boston, MA USA; 7 Dicerna Pharmaceuticals, Inc., Lexington, MA USA; 8 Informatics Institute, School of Medicine, University of Alabama at Birmingham, Birmingham, AL USA; 9 Division of Hematology/Oncology, Vanderbilt University Medical Center, Nashville, TN USA

**Keywords:** Cancer metabolism, Chronic myeloid leukaemia

## Abstract

Leukemic stem cells (LSCs) can acquire non-mutational resistance following drug treatment leading to therapeutic failure and relapse. However, oncogene-independent mechanisms of drug persistence in LSCs are incompletely understood, which is the primary focus of this study. We integrated proteomics, transcriptomics, and metabolomics to determine the contribution of STAT3 in promoting metabolic changes in tyrosine kinase inhibitor (TKI) persistent chronic myeloid leukemia (CML) cells. Proteomic and transcriptional differences in TKI persistent CML cells revealed BCR-ABL-independent STAT3 activation in these cells. While knockout of STAT3 inhibited the CML cells from developing drug-persistence, inhibition of STAT3 using a small molecule inhibitor sensitized the persistent CML cells to TKI treatment. Interestingly, given the role of phosphorylated STAT3 as a transcription factor, it localized uniquely to genes regulating metabolic pathways in the TKI-persistent CML stem and progenitor cells. Subsequently, we observed that STAT3 dysregulated mitochondrial metabolism forcing the TKI-persistent CML cells to depend on glycolysis, unlike TKI-sensitive CML cells, which are more reliant on oxidative phosphorylation. Finally, targeting pyruvate kinase M2, a rate-limiting glycolytic enzyme, specifically eradicated the TKI-persistent CML cells. By exploring the role of STAT3 in altering metabolism, we provide critical insight into identifying potential therapeutic targets for eliminating TKI-persistent LSCs.

## Introduction

Drug-insensitivity is a significant problem in cancer therapeutics. While leukemic cells can acquire secondary mutations, in many instances, they activate alternative signaling pathways for survival and resist treatment without a mutational cause [1]. This non-mutational insensitivity could be due to transcriptional, epigenetic, or metabolic reprogramming of leukemic stem cells (LSCs) [1]. Chronic myeloid leukemia (CML) is an ideal disease to explore non-mutational mechanisms of drug-insensitivity. It is a clonal disorder originating from hematopoietic stem cells (HSCs) due to a single oncogenic fusion protein BCR-ABL, a constitutively active tyrosine kinase [2, 3]. The standard molecular targeted therapy for CML is tyrosine kinase inhibitors (TKI), which eradicates the bulk of the disease but not the quiescent LSCs [4–8]. Although most patients respond to treatment, few can discontinue treatment without disease recurrence due to these persistent drug-insensitive LSCs.

In CML, BCR-ABL phosphorylates Signal Transducer and Activator of Transcription 5 (STAT5), promoting cell survival and differentiation [9]. However, upon TKI-treatment, persistent cells have increased STAT3 activation [10, 11]. Genetic deletion of STAT3 with BCR-ABL overexpression impairs colony formation and CML initiation [12]. Moreover, the combination of a STAT3 small molecule inhibitor with TKI reduces the differentiation potential of TKI-persistent leukemic cells [11]. These studies suggest that STAT3 plays a vital role in TKI evasion of LSCs in a BCR-ABL independent manner; yet, the mechanism of STAT3 mediated TKI-persistence remains unknown. STAT3 is a transcription factor regulating genes for cell survival, proliferation and metabolism [13, 14]. Interestingly, STAT3 can also enter the mitochondria to mediate survival during cellular stress [15–17].

Combining traditional treatment strategies with metabolic inhibitors can eradicate LSCs [18, 19]. For instance, targeting amino acid metabolism, fatty acid oxidation, or glutaminolysis along with the standard treatment eliminates AML LSCs [19–21]. While in T cell acute lymphoblastic leukemia, treatment modifies cellular metabolism from glutaminolysis to glycolysis to evade drug-induced cellular stress. One glycolytic enzyme important in cancer is pyruvate kinase M2 (PKM2) that provides a survival advantage to diseased cells and plays a role in drug resistance [22, 23]. Significantly in CML, PKM2 deletion constrains disease progression and prolongs survival [24].

This study investigates the mechanism of STAT3-mediated TKI evasion in CML. We show that TKI-persistent LSCs undergo STAT3 dependent transcriptional and metabolic changes from oxidative phosphorylation to glycolysis. These insights led to the discovery that TKI-persistent LSCs are susceptible to STAT3 inhibition and disruption of glycolysis. These findings reveal how STAT3 enables TKI-persistent CML cells to evade drug treatment in an oncogene-independent manner and provides a therapeutic window to target these cells specifically.

## Material and methods

### Mouse models

All mice are housed in UAB’s animal facility and experiments performed under IACUC approved protocol. Double transgenic BCR-ABL×SCL-tTA mice were used as a CML model at ~60% myeloid cells in peripheral blood, tested by flow cytometry as well as HemaVet [25, 26]. STAT3^flox/flox^ mice (Jackson Laboratory) were bred with BCR-ABL×SCL-tTA mice and MxCre mice to generate MxCre×STAT3^flox/flox^×CML mice (CML-STAT3 mice). H2b-GFP-CML mice were generated by breeding BCR-ABL×SCL-tTA mice to Rosa^26+/rtTA^ Col1A1^+/TetOP-H2B-GFP^ homozygous mice (Jackson Laboratory) [27]. Males and females were equally distributed throughout the study.

### Bone marrow transplant

8-week-old CD45.1 mice were sub-lethally irradiated (450 rads), and retro-orbitally transplanted with 5 × 10^6^ bone marrow cells from CD45.2 CML or control mice. Imatinib treatment was started ~4 weeks post-transplant [26]. For both the primary and transplanted mice, 200 or 400 mg/kg imatinib was administered by gavage every other day for 30 days. For STAT3 excision, 100 mg/kg pI:pC was administered i.p. every other day for 12 days after the mice showed sign of disease (myeloid expansion detected in the peripheral blood) [26]. For STAT3 small molecule inhibition studies, mice were treated with 25 mg/kg LLL12 i.p every alternate day in combination with imatinib for 30 days.

### ChIP-seq

Cells were cross-linked and sheared for ChIP with IgG and pSTAT3-Y705 (#9131, Cell Signaling). Double stranded cDNA libraries were constructed using Index Illumina library construction and sequenced [28]. Analysis of ChIP-seq: Optical duplicates were removed from raw fastq files using clumpify from BBMap (https://sourceforge.net/projects/bbmap/) with the flags “dedupe spany addcount”. This was followed by, adapter trimming using BBDuk (from BBMap) with the flags “ref = /…/bbmap/resources/adapters.fa ktrim=l hdist=2”. Next, reads were trimmed using trimmomatic [29] with the flags “LEADING:20 SLIDINGWINDOW:4:20 TRAILING:20 MINLEN:20”. After cleanup, reads were aligned to mm10 using bwa mem v0.7.17-r1188 [30] with default settings. Bam file sorting and indexing was performed using samtools [31]. Bam coverage maps were generated using bamCoverage from deeptools [32] using default. These coverage maps are uploaded to GEO for access. Next, peak calling was performed on all samples using MACS2 [33], HOMER [34], Genrich (available at https://github.com/jsh58/Genrich) and SICER2 [35, 36]. All peak callers were run in default parameters for broad peaks as recommended by developers. Once peaks were called, they were merged using HOMERs mergePeaks command with the flag “-d 100.” Average fold change of each peak over input was calculated using a custom python script and considering only uniquely mapped reads. Peaks were also annotated using HOMER’s annotatePeaks command using the mm10 genome. To ascertain peak overlap between the 3 CML/TKI/WT samples, HOMERs mergePeaks command was used with the flag “-d given”, and resultant sub-peak files annotated using HOMER’s annotatePeaks command with default parameters. Genome coverage plots were generated using ngsplot [37] with the flags “-G mm10 -R tss -L 2000 -RB 0.05. The ChIP-seq enrichment plot was generated using plotFingerprint from the deepTools suite with the flags “-skipZeros -bs 25.” For generating coverage regions presented within the manuscript, normalized bam coverage maps were generated using bamCoverage using the flags “-outFileFormat bedgraph -normalizeUsing None -binSize 25 –effectiveGenomeSize 2652783500.” The generated bedgraphs were then imported into R v4.0.3 (available at https://www.r-project.org/index.html) and figures generated using the package Sushi [38].

### RNAseq

RNA was isolated using the RNeasy Plus mini kit (Qiagen). Libraries were prepared with NexteraXT library construction (Illumina). The quality and size of the indexed libraries were determined using the BioAnalyzer (Agilent) and sequenced. Analysis was performed using Partek^®^ Flow^®^ software. Briefly, double-ended sequencing reads were aligned to the mouse (mm10) or human (hg38) using Spliced Transcripts Alignment to a Reference (STAR 2.6.1d). Aligned reads were then quantified to the transcriptome (RefSeq Transcripts 92) and normalized. Identified differentially expressed genes were used for hierarchal clustering and pathway analysis.

### Metabolomics and stable isotype tracing

Metabolomics assays were performed via ultra-high pressure-liquid chromatography-mass spectrometry (Vanquish and Q Exactive, Thermo Fisher) using the 3 min method [39]. For stable isotope tracing, K562 cells were incubated with ^13^C_3_-1,2,3-glucose (Sigma Aldrich) or ^13^C_5_^15^N_2_-L-Glutamine (Cambridge Isotope Laboratories) or U-^13^C_16_-Sodium Palmitate (Sigma Aldrich) for 30 min to 72 h where indicated [20]. From each experiment, the labeled isotopologues as a percentage of the total (labeled + unlabeled) were calculated, normalized to the relative abundance of each substrate. Data was analyzed using metaboanalyst [40].

### Statistical analysis

Analyses were performed depending on the spread of the variable and reported as standard deviation (SD). A Shapiro–Wilk test determined normal versus abnormal distributions, and all continuous variables were tested for mean differences. Depending on the spread of variable both nonparametric: Mann–Whitney *U* test, ANOVA Kruskal–Wallis test, Wilcoxon test, and parametric: Student’s *t* test and ANOVA were used. For ANOVA, Tukey’s or Sidak post-test was used to compare groups (GraphPad Prism version 7.0, La Jolla, CA).

### Data sharing statement

The data are deposited in NCBI’s Gene Expression Omnibus, accessible through GEO series accession number GSE152713.

## Results

### TKI-persistent CML cells have a distinct STAT3 mediated transcriptional and proteomic profile

**Fig. 1:**
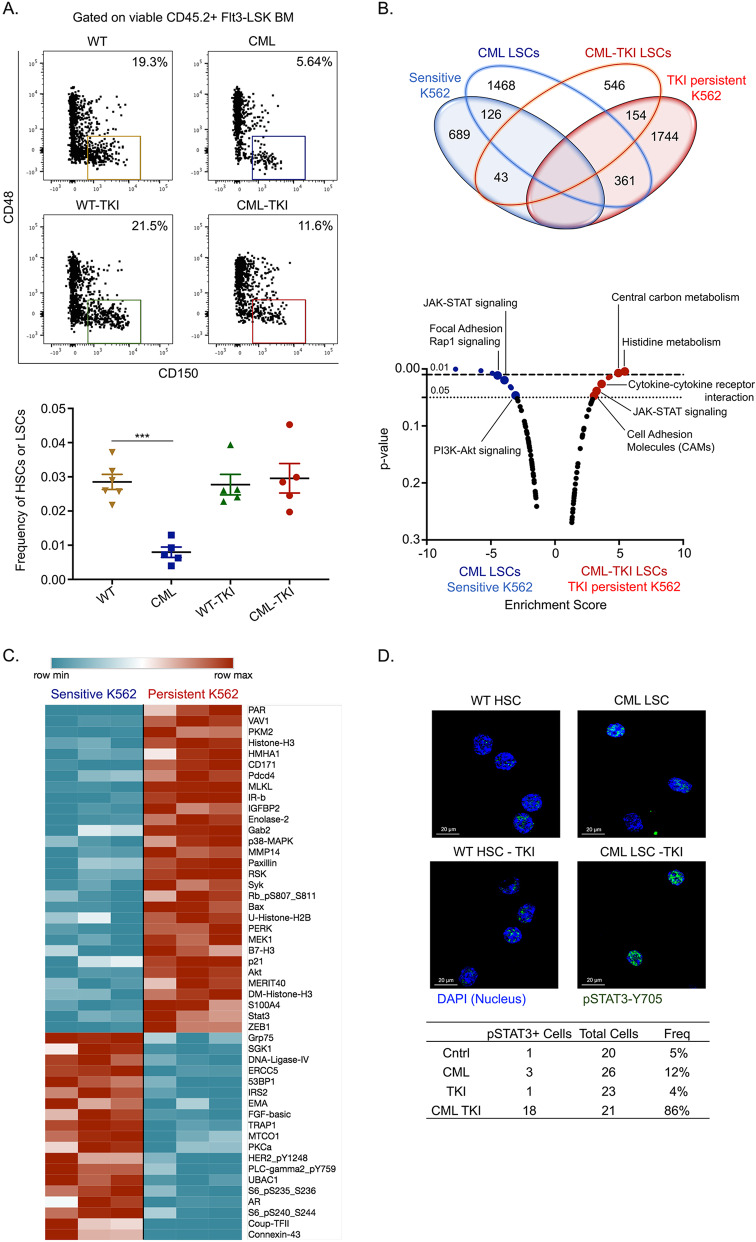
TKI-persistent CML LSCs and K562s have a distinct transcriptional and proteomic signature. **A** Representative FACS plot (left) and scatter plot (right) for frequency of HSC/LSC (LSK Flt3^−^CD48^−^CD150^+^) off the stem and progenitor (LSK Flt3^−^) cells in the BM of CML, CML mice treated with imatinib (200 mg/kg) for 4 weeks and their respective controls. *n* = 5. **B** Venn diagram (top) and KEGG pathway analysis (bottom) for common genes upregulated in LSCs obtained from CML mice and CML mice treated with imatinib (200 mg/kg) for 4 weeks and sensitive and IM-persistent K562 cells, obtained from RNA-seq. **C** Heatmap depicting differentially expressed protein in sensitive and persistent K562 with a fold change >1.4 and *p* value ≥ 0.01 when assayed by Reverse Phase Protein Array (RPPA). **D** Representative confocal imaging of (HSC/LSC (Lin^−^cKit^+^Sca1^+^Flt3^−^CD150^+^CD48^−^) for pSTAT3-Y705 (Green) and nucleus (Blue). Frequency of cells having stat3 expression is shown in the table to the right. The transcriptomic and proteomic data was carried out in triplicates. For scatter plot, two-way ANOVA was used to determine statistical significance with Tukey’s multiple comparison. *p* values < 0.05 were considered statistically significant. *p < 0.05, **p < 0.01, ***p < 0.001, ****p < 0.0001; ns = not significant.

TKIs reduce disease burden in patients, and yet, upon drug withdrawal, the disease recurs [41, 42] due to the persistence of TKI-insensitive LSCs [4–7]. To mimic this phenotype, we used a previously described transgenic CML mouse model [25]. In brief, once the mice are taken off tetracycline, the oncogene BCR-ABL gets expressed in the stem cells, giving rise to disease with myeloid expansion detected after 4 weeks [25, 26]. TKI treatment, in this case Imatinib (IM), was carried out for 4-weeks after the mice had a leukemic burden of 40% [26]. Similar to patients, IM-treated CML mice had reduced disease burden with the persistence of LSCs, which phenotypically resemble HSCs (Figs. 1A and S1A) [43, 44]. One proposed mechanism for the survival of CML LSCs with TKI-treatment is quiescence [7]. To determine the quiescence of LSCs, we used a pulse and chase H2b-GFP mouse model, wherein GFP is lost with each cell division leaving dormant cells marked over time [27]. H2b-GFP-CML mice revealed that IM-persistent LSCs retained more GFP, which was more quiescent than untreated LSCs (Fig. S1B). Transcriptomic analysis of these untreated and IM-persistent cells in vivo and in vitro revealed significant differences in gene expression as well as enriched pathways (Fig. S1C, D). We focused on signatures common between our in vitro and in vivo model by integrating our murine LSC and K562 RNA-seq data (Fig. 1B). Pathway analysis on these common, differentially upregulated genes revealed that the CML cells (126 genes) had enriched Rap1 and PI3-Akt signaling pathway, while the IM-persistent cells (154 genes) had enrichment in the central carbon metabolism, histidine metabolism and cell-cell interaction pathways (Fig. 1B). This is consistent with previous findings demonstrating BCR-ABL mediated regulation of FOXO, Ras/MAPK, PI3K/AKT/mTOR, Wnt, and STAT5 signaling [3, 43]. However, targeting these pathways in combination with TKI, failed to eradicate LSCs suggesting alternative mechanisms of cell survival [3, 8]. Therefore, we sought to identify additional signaling pathways contributing to transcriptional changes in IM-persistent CML cells by performing reverse-phase protein array. Active signaling proteins in the IM-persistent cells included STAT3, AKT1, MAP2K1, and PKM2 (Fig. 1C; Table S1). Pathway analysis of the top differentially activated proteins contributed to glycolysis, HIF and FoxO signaling in IM-persistent K562 (Fig. S1E, F). String analysis of the differentially active proteins within IM-persistent K562 revealed STAT3 and AKT1 as central nodes (Fig. S1G) [45]. Increased STAT3 activation was also observed in our murine CML model, where 86% of IM-persistent LSCs having pSTAT3-Y705 as opposed to 12% of untreated LSCs (Fig. 1D). Overall, these data indicate that the IM-persistent CML LSCs and K562 are transcriptionally rewired and have distinct proteomic signatures suggestive of metabolic changes.

### STAT3 is essential for the leukemic potential of TKI-persistent LSC

**Fig. 2:**
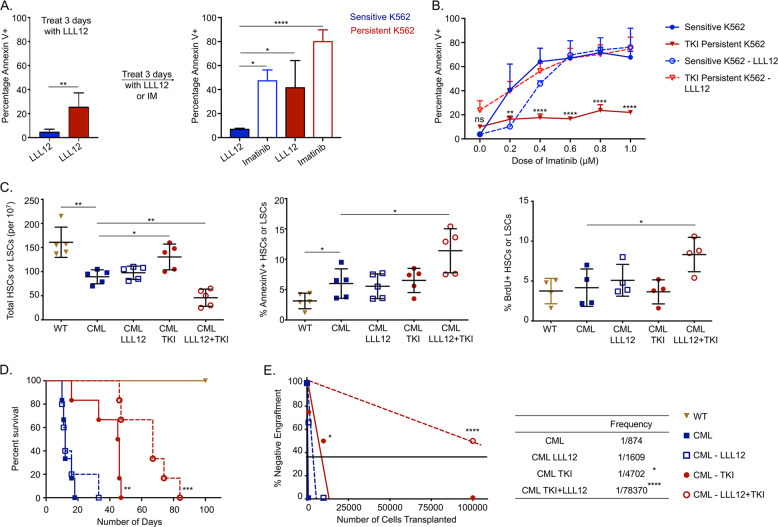
Active STAT3 is essential for survival and leukemic potential of TKI-persistent CML LSCs. **A** The sensitive and persistent K562 were treated with LLL12 (1 µM) for 3 days. Treatment was switched to either imatinib (1 µM) or continued with LLL12 (1 µM) for additional 3 days. Cells were analyzed for AnnexinV by flow cytometry. **B** Dose curve of imatinib for the sensitive and persistent K562 untreated or treated with LLL12 (1 µM) for 3 days. The cells were stained with annexin V and analyzed by flow cytometry. **C** HSC or LSC count per 10 million BM cells (left), percent AnnexinV stem cells (middle) and 24 hr BrDU incorporation in stem cells (right) of control and CML mice post 4 weeks of imatinib (200 mg/kg) or LLL12 (25 mg/kg) treatment individually or in combination. *n* = 5. **D** Survival curves of control and CML mice post 4-week imatinib (200 mg/kg) or LLL12 (25 mg/kg) treatment alone, or in combination. **E** Limiting dilution CRU (competitive repopulation unit) assay is shown. The indicated numbers of control or leukemic-exposed HSCs were transplanted along with 2 × 10^5^ BM cells of a competitor (CD45.2^+^) into lethally irradiated hosts (CD45.1^+^). Reconstitution was evaluated in the blood at 16 weeks post-transplantation (*p* = 0.0001). Mice with CD45.1^+^ chimerism <0.3 were considered non-responders. The data is a representative or pool of mean ± SD from two to three independent experiments. Unpaired student *t* test or Two-way ANOVA was used to determine statistical significance with Tukey’s multiple comparison. *p* values < 0.05 were considered statistically significant. *p < 0.05, **p < 0.01, ***p < 0.001, ****p < 0.0001; ns = not significant.

Since STAT3 and AKT were central nodes driving differential signaling in TKI-persistent K562, we evaluated their potential as therapeutic targets. Inhibition of AKT has previously been demonstrated to increase proliferation and apoptosis of CML LSCs but it also impairs maintenance, self-renewal and quiescence of normal HSCs, making it a difficult target [46, 47]. Alternatively, STAT3 has been essential for initiating disease and TKI resistance in CML in an oncogene-independent manner [10–12]. Consistently, we observed reduced pBCR and pSTAT5, along with increased pSTAT3-Y705 but not pSTAT3-S727 and pCRKL in IM-persistent K562s (Fig. S2A) [10, 11]. STAT3 CRISPR knock-out sensitive K562s had reduced cell growth and survival when treated with increasing doses of IM, indicating the importance of STAT3 for CML persistence during IM-treatment (Fig. S2B, C). To test the impact of STAT3 KO on LSCs in vivo, we generated MxCre×STAT3^flox/flox^ × CML (CML-STAT3) mice. Conditional deletion of STAT3 from hematopoietic cells of CML mice after disease progression significantly increased LSCs and reduced leukemic burden, suggesting the importance of STAT3 in CML maintenance (Fig. S2D–F). As STAT3 KO eliminates the pro-inflammatory IL-6 signature necessary for the maintenance of CML disease burden [26], it precluded our ability to use a genetic loss of STAT3 for studying IM-persistent LSCs. To circumvent this problem, we used a direct inhibitor of STAT3 phosphorylation at the Y705 site, LLL12 (Fig. S2G), unlike the widely used JAK2-related STAT3 inhibitors, WP1066 or Ruxolitinib [48, 49]. Pre-treatment of K562 cells with LLL12 followed by IM treatment led to apoptosis of 80.4 ± 9.4% IM-persistent K562 (Fig. 2A). Moreover, pSTAT3-Y705 inhibition also significantly increased persistent cells’ susceptibility to IM in a dose-dependent manner with minimal effect on the sensitive K562 (Fig. 2B). Consistently, in vivo LLL12 treatment alone had no impact on LSC count; however, dual administration of LLL12 and IM significantly reduced LSC number in CML mice (Fig. 2C). This reduction was associated with significantly increased apoptosis and proliferation of dual treated LSCs compared to LSCs from mice treated with IM or LLL12 alone (Fig. 2C). Furthermore, LLL12/IM dual treatment extended survival of CML mice compared to single-agent treated and untreated CML mice (Fig. 2D). Moreover, the dual treated LSCs had reduced engraftment upon transplantation (Fig. 2E). These data thus demonstrate that STAT3 is essential for the maintenance and leukemogenic potential of TKI-persistent LSCs.

### TKI-persistent LSCs are transcriptionally rewired with differential STAT3 localization to metabolic genes

**Fig. 3:**
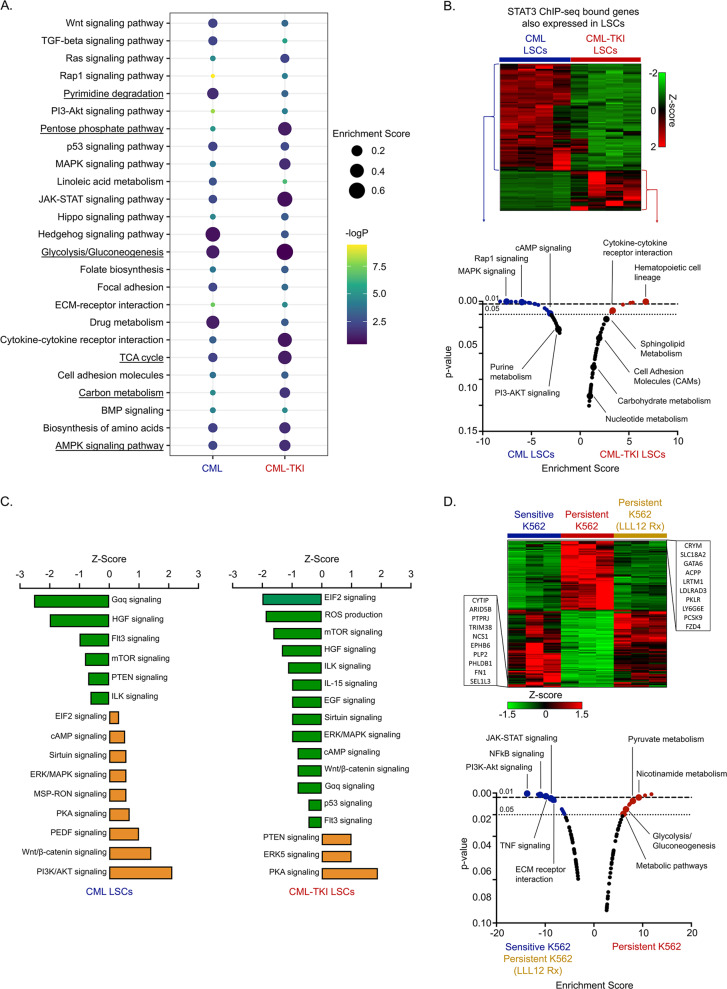
STAT3 localizes to metabolic genes in transcriptionally altered TKI-persistent LSCs. **A** Kegg GO Pathway analysis depicting *z* score and *p* value for the genes differentially bound by pSTAT3-Y705 in the CML and IM-persistent CML LSK. **B** Heatmap and pathway analysis for integration of the pSTAT3-Y705 bound ChIP-seq peaks and the upregulated RNA-seq genes in the CML and IM-persistent LSK and LSCs respectively. **C** Ingenuity Pathway analysis bar chart depicting *z* scores of pathways correlated to overlapping genes of CML vs WT bulk-RNA sequencing and pSTAT3-Y705 ChIP-seq peaks unique to CML as well as overlapping genes of CML-TKI vs WT bulk-RNA sequencing and pSTAT3-Y705 ChIP-seq peaks unique to CML-TKI. **D** Heatmap and pathway analysis for upregulated genes common between sensitive K562 and IM-persistent K562 treated with 1 µM LLL12 (1 µM) but different from IM-persistent K562. The transcriptomic data were carried out in triplicates.

STAT3 targeting agents have not yet progressed beyond clinical trials’ initial stages due to lack of specificity, unpredictable pharmacokinetics, and toxic effects [50]. These challenges highlight the need to identify the molecular mechanism of drug-insensitivity downstream of STAT3 for alternative therapeutic targets. Since active STAT3 is a transcription factor [51], we performed pSTAT3-Y705 ChIP-seq using Lin^−^Sca1^+^cKit^+^ (LSK) cells to determine the STAT3 transcriptional targets in IM-persistent CML stem and progenitors. We observed 13444 unique pSTAT3-Y705 localizations from IM-treated CML mice compared to 9931 in control and 10266 sites in leukemic mice (Fig. S3A). Moreover, motif analysis revealed that unique sites of enrichment from IM-persistent CML LSK had a greater percentage of peaks with the known STAT3 consensus sequence compared to WT and CML (Fig. S3B). Pathway analysis on these pSTAT3-Y705-bound sites revealed target genes enriched in glycolysis, carbohydrate metabolism, pentose phosphate pathway, AMPK signaling and cytokine-cytokine interaction in the CML-TKI LSK, while hedgehog signaling, Wnt/β-catenin signaling and TGFβ signaling are enriched in the CML LSK (Fig. 3A), corroborating the enriched pathways observed in the transcriptomic analysis. Integration of ChIP-seq and RNA-seq analysis revealed that genes bound by pSTAT3-Y705 as well as those upregulated in CML-TKI LSCs were also found to be enriched in cytokine interaction and metabolism related signatures (Fig. 3B). Moreover, ingenuity pathway analysis also disclosed significant positive correlation of PKA signaling in the CML-TKI LSCs, which is known to be activated by glycolysis [52] (Fig. 3C). Hence, to specifically define STAT3 mediated transcriptional changes, we performed RNA-seq on sensitive, IM-persistent and LLL12 treated IM-persistent K562. Since STAT3 inhibition sensitizes the IM-persistent K562s (Fig. 2B), we focused on signatures shared between LLL12 treated persistent and sensitive cells (Fig. 3D). Pathway analysis revealed an enrichment of pyruvate metabolism and glycolysis in IM-persistent K562 (Fig. 3E). Meanwhile, the sensitive and LLL12 treated cells were enriched in the NFkB, PI3-Akt, TNF, and JAK-STAT signaling pathway known to be essential for CML maintenance (Fig. 3E) [3]. Overall, these analyses reveal STAT3 mediated transcriptomic changes in TKI-persistent CML cells with enrichment of metabolic pathways.

### Drug insensitive CML cells have altered metabolism mediated by STAT3

**Fig. 4:**
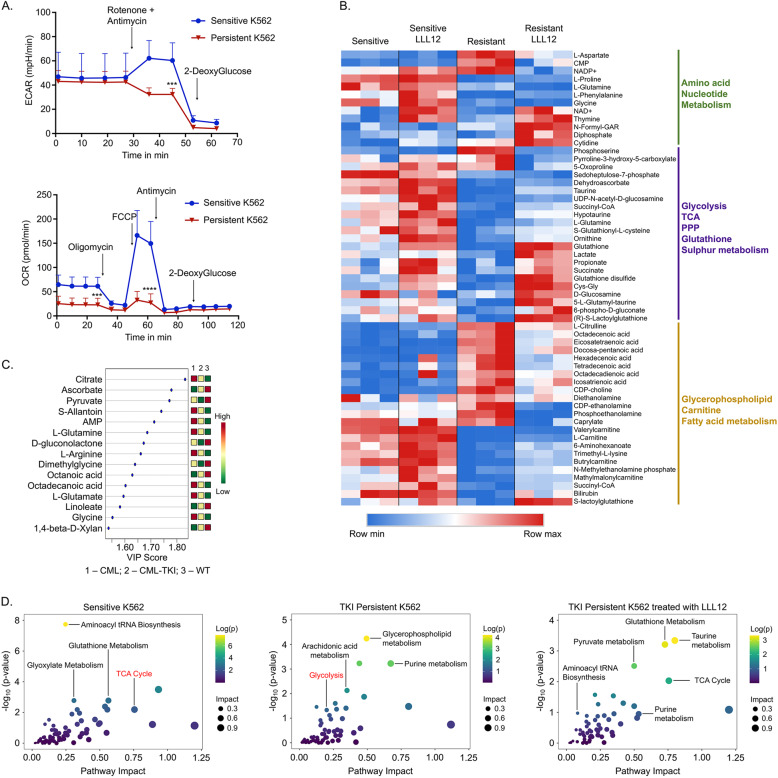
STAT3 contributes to altered metabolism in TKI-persistent CML cells. **A** ECAR (top) from glycolysis rate assay (GRA) and OCR (bottom) from mitochondrial stress test (MST) of 75,000 sensitive and IM-persistent K562 measured by seahorse. **B** Heatmap for the top 50 differentially expressed metabolites between IM-persistent K562 and those treated with LLL12 (1 µM) with a *p* value ≤0.05. Raw values were used to generate the heatmap. **C** VIP plot for component 1 of the PLS-DA comparing WT HSCs, CML LSCs, IM-persistent CML LSCs. The VIP score indicates the contribution of the metabolite to the clustering pattern. **D** Scatter plots obtained from integration of the K562 metabolic profile with the K562 RNA-seq data. All figures are mean ± SD of a representative data of at least 2–3 independent experiments. Unpaired student *t* test. was used to determine statistical significance with Tukey’s multiple comparison. *p* values < 0.05 were considered statistically significant. *p < 0.05, **p < 0.01, ***p < 0.001, ****p < 0.0001; ns = not significant.

CML stem and progenitor cells rely on oxidative phosphorylation compared to their non-transformed counterparts [44, 53]. Moreover, short-term TKI-treatment of sensitive cells reduces glucose uptake and its utilization for nucleotide and fatty acid synthesis while increasing mitochondrial activity [54, 55], but alterations in the metabolism of IM-persistent CML cells is yet to be studied. Thus, to examine metabolic differences between sensitive and IM-persistent K562, we measured cellular mitochondrial function and glycolysis using extracellular flux analyzer. We observed similar basal extracellular acidification rate (ECAR) but reduced reserve glycolytic capacity of the persistent K562s compared to sensitive cells indicating maximal glycolysis usage in persistence (Fig. 4A). Moreover, oxygen consumption rate (OCR) was significantly reduced in IM-persistent cells (Fig. 4A), and hence we next evaluated mitochondrial damage. We observed three times more total mitochondrial DNA in IM-persistent K562 (Fig. S4A); however, there was no difference in mitochondrial counts or area (Fig. S4B). Furthermore, we noted reduced protein abundance of complex III and IV, Cytochrome C reductase and oxidase components of the electron transport chain (ETC) (Fig. S4C) [56]. These data indicate loss of mitochondrial metabolism and function with a maintained glycolytic rate in IM-persistent K562, which suggests reduced energy requirement observed with dormancy (Fig. S1B).

STAT3, mostly pSTAT3-S727, acSTAT3-K685 and unphosphorylated STAT3 play an essential role in maintaining mitochondrial function by localizing to the inner mitochondrial membrane [16, 17, 57]. Mitochondrial STAT3 (mitoSTAT3) interacts with complex I, II, and IV of the ETC and also transcriptionally regulates mitochondrial DNA [58, 59]. We observed an accumulation of tSTAT3, pSTAT3-S727, and acSTAT3-K685 in the mitochondria of IM-persistent K562 compared to sensitive K562s (Fig. S4D). Hence, to determine STAT3 mediated alteration of metabolism in IM-persistent CML cells, we generated metabolic profiles with STAT3 inhibition. We noted an accumulation of nucleosides and unsaturated fatty acids in IM-persistent K562, which reduced with STAT3 inhibition (Fig. 4B). In addition, stable metabolic profiles on murine LSCs and control HSCs with and without IM-treatment also identified metabolic changes (Fig. S4E). We noted reduced metabolite abundance in IM-persistent murine LSCs (Fig. S4E) consistent with reduced metabolic activity seen with IM-persistent K562. Principal component analysis revealed that LSCs have a unique metabolic profile compared to HSCs, while IM-persistent LSCs are closer to their healthy counterparts (Fig. S4F). Further analysis showed reduced Component 1 metabolites (part of TCA cycle entry) in IM-persistent LSCs (Fig. 4C). Integrative principal component analysis of K562s and murine LSCs show that the IM-treated samples cluster together (Fig. S4G). Moreover, integrative pathway analysis of upregulated genes and enriched metabolites revealed that similar to sensitive K562, IM-persistent K562 treated with STAT3 inhibitor had an enriched TCA cycle metabolic pathway while IM-persistent K562 had an enriched glycolytic pathway (Fig. 4D). Because metabolic profiling and RNA-seq provide a snap-shot, we validated the findings with seahorse experiments as a functional readout. We observed that STAT3 inhibition did not impact OCR (Fig. S4H) and although it led to a non-significant increase in the basal ECAR, the compensatory glycolysis of IM-persistent K562 increased to be similar to the sensitive K562s (Fig. S4H). Overall, these data suggest that STAT3 leads to metabolic alterations in TKI-persistent LSCs towards a reduced mitochondrial activity state while maintaining energy production through glycolysis.

### TKI-insensitive CML cells are dependent on glycolysis

**Fig. 5:**
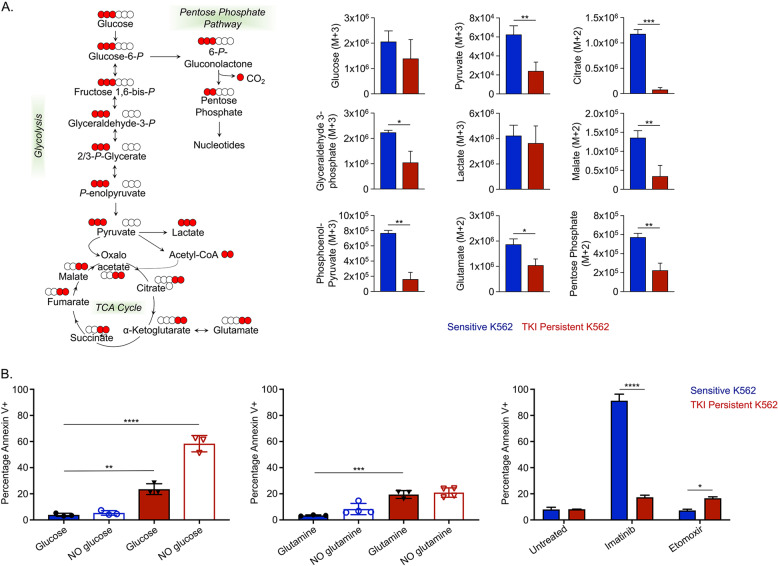
TKI-persistent K562 have an active glycolysis and increased fatty acid accumulation. **A** Unit area plots for metabolites labeled in glycolysis, citric acid cycle and PPP post 3 h incubation of 200,000 sensitive and IM-persistent K562 with isotopically labeled ^13^C_1,2,3_-glucose. The metabolites were measured with UHPLC-MS. **B** Sensitive and IM-persistent K562 treated with 1 µM Imatinib were starved with glucose (right) or glutamine (middle) for 48 h or treated with 10 µM Etomoxir for 3 days. Cells were analyzed by flow cytometry using annexinV apoptosis assay. The data are representative mean ± SD from two independent experiments. Unpaired student *t* test or Two-way ANOVA was used to determine statistical significance with Tukey’s multiple comparison. *p* values < 0.05 were considered statistically significant. *p < 0.05, **p < 0.01, ***p < 0.001, ****p < 0.0001; ns = not significant.

LSCs utilize multiple pathways to evade drug treatment, including dependency on amino acid metabolism, fatty acid oxidation or using glutamine as a carbon source [19–21]. To determine contributions of metabolic substrates in sensitive and IM-persistent K562, we carried out metabolic flux analysis using 1,2,3-^13^C_3_-Glucose, ^13^C_5_^15^N_2_-Glutamine and U-^13^C_16_-Palmitate. Glucose tracing revealed reduced labeling of several glycolytic metabolites in IM-persistent K562 (Fig. 5A). Despite having a slower flux of ^13^C-Glucose through glycolysis in IM-persistent K562, the intracellular level of labeled lactate was comparable with the sensitive K562 (data not shown). Moreover, sensitive K562 had a higher flux of glucose-derived carbon atoms into the TCA cycle (Fig. 5A), consistent with a higher OCR (Fig. 4A). Furthermore, IM-persistent K562 had lower levels of glucose-derived labeled carbons into pentose phosphate pathway, or PPP-derived ATP and ^13^C-acetyl-carnitine, consistent with acetyl-CoA (Fig. S5A), an entry metabolite of TCA cycle. These data combined with reduced levels of ^13^C-Glucose derived ATP in IM-persistent K562 (Fig. S5A) suggest decreased oxidative phosphorylation. Importantly, stable metabolite profiles revealed an increase in α-Ketoglutarate to Citrate ratio (Fig. S5B), suggesting a block in the TCA cycle beyond α-Ketoglutarate and an increase in reductive carboxylation as a result of the altered mitochondrial function [60, 61]. To identify alternative carbon sources preferentially utilized by IM-persistent K562, we carried out glutamine metabolic flux analysis. Consistent with glucose tracing, labeled carbon incorporation from glutamine into TCA cycle was reduced in IM-persistent K562 (Fig. S5C) along with increase in citrate isotopologues M + 5 (reductive carboxylation) to M + 4 (oxidative) ratio (Fig. S5D). In line with the role of reductive carboxylation as fuel to fatty acid synthesis [60], we observed an increase in glutamine-derived ^13^C-acetyl-carnitine in IM-persistent K562 (Fig. S5D). Palmitate tracing highlighted an increase in fatty acid elongation and desaturation in IM-persistent K562 (Fig. S5E), which corroborates the accumulation of unsaturated fatty acids observed in steady-state analyses (Fig. 4B). Moreover, palmitate tracing revealed palmitate oxidation as a source of acetyl-carnitine in IM-persistent K56s, further utilized to make significantly more citrate (Fig. S5E). Surprisingly, treatment with the FAO inhibitor, Etomoxir, had a negligible impact on cell survival of IM-persistent K562 (16.65 ± 1.06%) (Fig. 5B). However, starving IM-persistent K562 of glucose led to increased apoptosis, 58.37 ± 6.23% (Fig. 5B), indicating their dependence on glucose for survival but not fatty acids or glutamine (Fig. 5B). Overall, these results indicate that TKI-persistent K562 accumulate fatty acids but depend on glucose for their survival.

### Glycolysis is important for survival of TKI-persistent CML cells

**Fig. 6:**
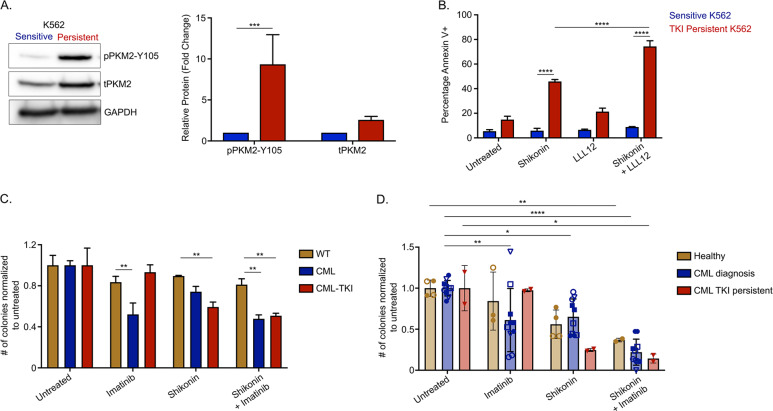
Glycolysis is important for the survival of TKI-persistent CML stem and progenitor cells. **A** Western blot for PKM2 using sensitive and IM-persistent K562 cell lysate. The bar plot represents density of the persistent protein bands relative to sensitive and normalized to the housekeeping protein, GAPDH. **B** Sensitive and IM-persistent K562 analyzed for apoptosis via flow cytometry post 3-day treatment with IM (1 µM), LLL12 (1 µM), or Shikonin (0.2 µM) alone, or in combination. **C** Colony-forming assay for 1000 LSK cells/mL sorted and plated from control, CML, and imatinib (400 mg/kg) treated CML mice. The plates were treated with IM (1 µM) or Shikonin (0.5 µM) alone, or in combination, incubated in hypoxia and colonies counted after 10 days. Data is represented as counts normalized to untreated. **D** Colony-forming assay for 1000 Lin^-^CD34^+^ BM cells/mL sorted and plated from 2 healthy individuals, 5 CML patients at diagnosis and 1 IM treated disease persistent patient. The plates were treated with Imatinib (1 µM) or Shikonin (0.5 µM) alone, or in combination, incubated in hypoxia and colonies counted after 10 days. Data are represented as counts normalized to untreated. The data are representative or a pool of mean ± SD from 2 to 3 independent experiments. Unpaired student *t* test or Two-way ANOVA was used to determine statistical significance with Tukey’s multiple comparison. *p* values < 0.05 were considered statistically significant. *p < 0.05, **p < 0.01, ***p < 0.001, ****p < 0.0001; ns = not significant.

STAT3 activation leads to transcriptional and metabolic changes towards glycolysis and is critical for the survival of TKI-insensitive cells. Additionally, glycolysis modulates quiescence (Fig. S1B) by reducing energy demand [62]. From our LSC RNA-seq, we observed that genes in the glycolysis/gluconeogenesis pathway are more enriched in the IM-persistent LSCs (Fig. S6A). Treating IM-persistent K562 with 2-Dexoyglucose, 2-DG, a competitive glycolysis inhibitor, led to increased apoptosis (Fig. S6B), consistent with glucose starvation (Fig. 5B). However, since 2-DG cannot be translated clinically, we looked at other glycolytic targets. Interestingly, we observed approximately threefold increase in PKM2 and approximately tenfold increase in pPKM2-Y105 in IM-persistent K562s (Figs. 1C and 6A). PKM2 in previous studies has shown to slow CML progression; however, it has importance in drug persistence is not known [24]. We hence used pharmacological inhibitors of PKM2, compound 3 K or Shikonin [63, 64], and observed selectively increased apoptosis of persistent K562s with dose escalation (Fig. S6C–F). Interestingly, dual treatment of IM-persistent K562 with LLL12 and Shikonin had an additive effect on apoptosis (Fig. 6B). Since in vivo shikonin treatment proved toxic for healthy mice (data not shown), we used ex vivo treatment to validate the K562 data in our murine model. Shikonin treatment of LSK leads to apoptosis of CML and IM-treated LSKs compared to the single and dual treated non-transformed LSK (Fig. S6G), consistent with PKM2 knock out of untreated CML cells [24]. In addition, we observed reduced colony-forming potential of IM-persistent LSK with Shikonin alone or in combination with IM (Fig. 6C). Similar trends were observed in colony-forming potential of shikonin-treated CD34^+^ cells from CML patients at diagnosis and following IM-treatment (Fig. 6D). Although, dual treatment of Shikonin and IM also reduced colony forming potential of non-transgenic CD34 + cells, it did not significantly affect the viability of these cells (Figs. 6D and S6H). Moreover, similar to K562 and murine LSK, we also observed reduced cell viability and increased apoptosis of CD34+ cells from IM-persistent CML patient samples treated with both IM and shikonin (Fig. S6H–I). These data indicate that IM-persistent LSCs have altered metabolism dependent on glycolysis for their survival, hence providing a target to eliminate the TKI-persistent LSCs in CML.

## Discussion

The persistence of cancer stem cells after drug treatment and subsequent relapse is a significant barrier to cure. Apart from mutational causes of drug resistance, cells can persist in drug treatment due to the survival of a drug-tolerant form [65]. In this study, we describe a non-mutational mechanism of STAT3-mediated transcriptional and metabolic regulation in drug-insensitive LSCs. Active STAT3 localized to several metabolic genes while also regulating mitochondrial metabolism. Notably, TKI-insensitive cells are reliant on glycolysis and the rate-limiting, stress-related enzyme PKM2. Understanding the mechanism of STAT3-mediated drug-insensitivity provides potential drug targets to eliminate these TKI-persistent cells and reduces the chances of relapse.

Given the importance of STAT3 in tumor growth, survival, and chemoresistance, several clinical trials have been carried out with STAT3 inhibitors. However, STAT3 targeting agents yield minimal success because they inhibit activators of STAT3, like JAK family members. Besides, STAT3 can be activated by other kinases explaining the survival of TKI-persistent CML cells treated with JAK-mediated STAT3 inhibitors [66]. Furthermore, many drugs that inhibit STAT3 activation bind the SH2 domain that shares homology to other STAT family members leading to toxic effects [50]. Recent drug discovery has been towards tagging STAT3 for ubiquitin-dependent degradation [67]. These agents hold some promise in clinical trials, yet understanding the downstream activity of STAT3 during TKI-persistence provides an additional opportunity to find alternative drug targets.

Consequently, we sought to understand the mechanism of STAT3 mediated drug persistence. We observed transcriptional adaptations of metabolic genes and found STAT3 localized to CPT1B and citrate synthase, critical enzymes for fatty acid oxidation and TCA cycle [14]. Conversely, STAT3 induces glycolysis in a hypoxic environment while reducing mitochondrial genes [68]. Interestingly, it can also enter mitochondria to regulate mitochondrial function [17, 58, 59], and cellular stress [15, 16]. These observations likely explained the increased mitoSTAT3 in TKI-persistent K562 with minimal mitochondrial activity and compensated by increased glycolysis. LSCs have an increased oxygen consumption rate upon initial drug treatment [53]; however, our data suggest that with prolonged treatment, the more quiescent, TKI-persistent cells are biased towards glycolysis, possibly due to reduced energy requirements. Additionally, reductive carboxylation, a de novo lipogenesis pathway [69], and elongation of fatty acids in TKI-persistent K562 are consistent with utilization of carbon from glucose for nucleic acid and fatty acid synthesis [54, 70]. Moreover, free fatty acids can form a feed-forward loop with fatty acid oxidation, a reserve energy source [71]. These indicate metabolic reorganization of TKI-persistent LSCs to aid survival under cellular stress.

The main regulator of cellular stress is PKM2, a key enzyme in glycolysis [63] that promotes reductive glutamine metabolism [61]. PKM2 knockout in healthy HSCs has shown no substantial defect minus loss of reconstitution potential observed upon multiple serial transplants. Additionally, PKM2 knockout delays CML onset and death [24], consistent with our data. Our findings uniquely reveal an increased dependence of TKI-persistent cells on PKM2. Oxidative stress can lead to PKM2 dimerization and phosphorylation to act as a transcription factor or kinase, with evidence of activating STAT3 [23, 72]. This observation suggests a possible feed-forward loop of PKM2 and STAT3 in regulating TKI-persistence and provides a potential drug target to eradicate the TKI-persistent LSCs.

These findings emphasize STAT3 regulation of gene expression and metabolism in TKI-persistent leukemic cells to a glycolytic state. Our study could be extrapolated to other drug-resistant diseases like Flt3-ITD AML, multiple myeloma or solid tumors that acquire oncogene independent drug-insensitivity. Additional work is needed to develop better therapeutic drugs for targeting STAT3, but we have characterized the downstream metabolic regulation as an alternative means to eliminate LSCs. Our findings implicate a therapeutic opportunity where inhibition of glycolysis along with TKI-treatment preferentially targets drug-insensitive LSCs. At the same time, non-transformed HSCs can survive due to activating alternative metabolic pathways. However, there is still an ever-increasing need for developing better and specific metabolic inhibitors, an impediment to clinical translation in cancer. Importantly, we have shown that integrating signaling pathways, transcriptomics and metabolomics can discover drug targets to eliminate treatment persistent cancer stem cells.

## Supplementary information

Supplement Material

Supplemental Table 1
