# Supplementary material for: Metabolic alterations mediated by STAT3 promotes drug persistence in CML
Source: Leukemia. Author manuscript; Available in PMC 2021 Dec 3. (PMC8632690; doi:10.1038/s41375-021-01315-0)
Supplement: Supplementary Methods and Figures [file NIHMS1716989-supplement-Supplementary_Methods_and_Figures.pdf]

SUPPLEMENTAL METHODS

Cell Culture: K562 lines were obtained from ATCC (CCL-243). The parental cells were made resistant to and maintained at 1μM imatinib (IM) as previously described (1). Briefly, the bulk culture of parental K562 cells (sensitive to IM) were treated with an incremental dose of IM starting at 0.2μM over the course of 8 weeks till the cells were resistant to 1.0μM IM. Experiments were carried out using the sensitive and persistent cells 2-3 times to show reproducibility and carry out statistical analysis.

Human samples: Samples were obtained from CML patients in chronic phase (CP) without prior imatinib treatment or persistent patients post imatinib treatment seen at the UAB Hospital. Healthy BM samples were obtained from AllCells. Samples were processed for $CD34^+$ cell selection with CliniMACS (Miltenyi Biotech, Teterow, Germany). Mononuclear cells were isolated by Ficoll-Hypaque (Sigma Diagnostics, St. Louis, MO) separation. $CD34^+$ cells were isolated by using immunomagnetic beads (Miltenyi Biotech, Auburn, CA). $CD34^+CD38^-$ cells were obtained by flow cytometry sorting as previously described (2). Sample acquisition was approved by the Institutional Review Board at UAB Hospital, in accordance with an assurance filed with and approved by the Department of Health and Human Services and met all requirements of the Declaration of Helsinki.

FACS staining and single-cell sorting: For FACS profiles, single-cell suspension was stained with a cocktail of monoclonal antibodies conjugated to fluorophores: Lineage markers: CD3 (17A2, PE-Cy5), CD19 (6D5, PE-Cy5), B220 (RA36B2), CD11b (ICRF44, PE-CY5), Gr-1 (RB6-8C5) and Ter-119 (TER-119, PE-Cy5), F4-80 (BM8, APC-Cy7); Progenitor markers: cKit (2B8, APC-Cy7), Sca1 (D7, APC), CD135 (A2F10, PE), CD48 (HM48-1, Pacific Blue), CD150 (SLAM, PE-Cy7), FcγRII/III (93, PE), CD41 (HIP8, FITC) and Endoglin (AFS98, Pacific Blue). DAPI was

used as a viability marker. LSR II with BD Diva Software was used to acquire data while FlowJo 10.4.2 (Tree Star) was used for analysis. For Cell Sorting, the whole bone marrow was depleted for Lineage marker positive cells using biotin and streptavidin beads on the autoMACS sorter. The lineage negative fraction was then stained(3); stem and progenitor cells were sorted using a FACSAria II (BD Biosciences).

Limiting Dilution Assay: Total bone marrow cells were sorted as $CD45.2^+$ from leukemic or treated mice 4 weeks following TKI and/or LLL12. Numbers of cells to be injected per mouse were sorted into individual wells of a 96-well plate containing 10^4 wild-type $CD45.1^+$ bone marrow (rescue cells). The contents of individual wells were injected into lethally irradiated $CD45.1^+$ recipients (600 rads, twice with a 3-hr interval). Peripheral blood was obtained from mice each month for at least 4 months for chimerism as previously described (4, 5).

Apoptosis Assay: Annexin V (ThermoFisher) with DAPI as a counter stain was used for the apoptosis assay. Cells were treated with the respective drugs alone or in combination for 6hrs, 24hrs, 3 days or 7 days. For 7 days, the media was changed on day 3. The cells were harvested and the standard protocol was followed for Annexin V assay. The samples were analyzed using flow cytometry.

Cell Cycle and Proliferation Assay: CML sensitive and resistant K562 cells were treated with their respective drug combinations for 3 days. The cells were then pulsed with 10µM EdU for 4hrs. The cells were then harvested, fixed, stained and analyzed according to the manufacturers protocol for Click-iT EdU flow cytometric assay kit (Invitrogen). For proliferation of murine HSCs and LSCs, BrdU was administered intraperitoneally (1mg/mouse), bone marrow cells were obtained 16 hr post-injection, and BrdU incorporation into stem and progenitor populations was analyzed as described before(6).

Colony Forming Assay: $Lin^{-}cKit^{+}Sca1^{+}$ were sorted from CML and IM treated CML mice and resuspended in MethoCult GF M3434 (Stem Cell Technology) or human $CD34^{+}$ cells or K562s were resuspended in MethoCult H4435 Enriched (Stem Cell Technology) with imatinib, LLL12 or shikonin, individually or in combination. Colonies were counted and images were taken after 10 days' incubation in hypoxia (human patient and murine) or normoxia (K562).

Western Blot and RPPA: Lysates were prepared as previously described (7). Blots were probed for pSTAT3-Y705 (D3A7 XP, Cell Signaling), pSTAT3-S727 (Cell Signaling), AcSTAT3-K685 (Cell Signaling), tSTAT3 (F-2, Santa Cruz), pSTAT5-Y694 (D47E7, Cell Signaling), tSTAT5 (D2O6Y, Cell Signaling), pBCR-Y177 (Cell Signaling), tBCR (Cell Signaling), pSRC-Y416 (Cell Signaling), tSRC (Cell Signaling), pPKM2-Y105 (Cell Signaling), PKM2 (D78A4, Cell Signaling), VDAC2 (Cell Signaling). The images were taken using a GBox imager. Densitometry analysis of the blots was carried using ImageJ and represented as relative density to the housekeeping gene B-Actin (C4, Santa Cruz) or GAPDH (14C10, Cell Signaling). Blots were quantified using Image J (1.52a Wayne Rasband, NIH, USA).

For RPPA, the samples were prepared and analyzed at MD Anderson Cancer Center as previously described (8-10).

STAT3 CRISPR: STAT3 knock out parental CML cells were prepared by CRISPR/Cas9 gene editing technology as previously described(11). Single-guide RNA targeting STAT3 was created by cloning appropriate primers into PX458 plasmid. sgRNA was designed to target exon 15 of the STAT3 DNA binding domain (GAGACCGAGGTGTATCACCA) using Benchling (Biology Software, 2020). The sgRNA cloned plasmid was transfected into the parental K562 cells using Nucleofector KitV (Lonza) electroporation according to manufacturer's instruction. Consequently,

GFP positive cells were sorted and single cell selection carried out for further validation and experiments.

Mitochondrial Isolation: Mitochondria was isolated from 20 million K562 cells using the Mitochondria isolation kit for cultured cells (Thermo Scientific). The mitochondrial pellet was then lysed with 2% Chaps. VDAC2 and B-Actin antibody were used for validating the purity of the mitochondrial and cytosolic fraction respectively.

Mitochondrial DNA copy number: DNA was isolated using the DNeasy Tissue kit (Qiagen). Quantitative real-time PCR was performed by using a SYBR Green master mix (Life Tech Corp). The primer sequences used for mtDNA were mtDNAF (5′-CACCCAAGAACAGGGTTTGT-3′) and mtDNAR (5′-TGGCCATGGGTATGTTGT TAA-3′). The primer sequences for the nuclear DNA were 18S-F (5′-TAGAGGGACAAGTGGCGTTC-3′) and 18S-R (5′-CGCTGAGCCAGTCAGTGT-3′) and targeted the human nuclear 18S DNA.
The mtDNA copy number was normalized to the amplification of the 18S nuclear amplicon, calculated as 2*(2^(mCt-nCt)).

Extracellular Flux Analysis: K562 cells were plated per well of an XFe96 plate coated with CellTak (Corning, 354240). Analysis of cellular bioenergetics was performed using the Seahorse XFe 96 Extracellular Flux Analyzer (Agilent). Glycolytic rate assay and Mito Stress test were performed according to the manufacturer's instructions (Agilent Technologies)(12, 13). Cell number and FCCP concentration were used based on the titration results for the different cell types.

TEM Imaging: K562 cells were fixed with 2% glutaraldehyde in 0.1M Cacodylate buffer followed by dehydration with 50% ethanol. The cells were then En bloc stained with 1% uranyl acetate in

50% ethanol for 30min in the dark. The stain was rinsed with 50% ethanol and then dehydrated to 100% ethanol thrice. Cells were infiltrated and embedded in Epon 812 resin. Thin sections were cut using a Leica EM-UC6 ultramicrotome and contrasted with uranyl acetate and Reynold's lead citrate. Images were taken using an FEI Tecnai-Spirit electron microscope operating at 80kV and an AMT Biosprint digital camera. Images were analyzed to calculate the mitochondrial area per cell using Image J (1.52a Wayne Rasband, NIH, USA).

Drug Preparation: For cell culture, 1mM of IM (obtained from LC laboratories, I55082G) working solution was prepared in warm sterile PBS, aliquoted and stored at -20. 1.5mg/mL stock solution of LLL12 (obtained from Biovision, 1792) was made using DMSO, diluted to 5mM using PBS and stored in -20. 50mM of Shikonin (obtained from Selleck Chemicals, S8279) stock solution was made in DMSO and stored in -80. 1mM working solution was made fresh every time before use with warm sterile PBS. For murine experiments, 1mg/µL IM was made in warm sterile PBS and stored in -20. On the day of treatment, the stock was further diluted to a dose of 200-400mg/200µL using PBS. 50mM LLL12 was made using DMSO, diluted to 5mM using PBS and stored in -20. On the day of treatment, the 5mM stock was diluted to inject 25mg/kg in 100µL of PBS.

SUPPLEMENTAL TABLE LEGEND

Table S1: RPPA normalized linear values for proteins detected in sensitive and IM-persistent CML cells with p-value <0.05

SUPPLEMENTAL FIGURE LEGEND

Figure S1: pSTAT3-Y705 is important to maintain resistance in the CML TKI insensitive cells

A: Representative FACS plot (left) and scatter plot (right) for frequency of myeloid cells (Gr1+CD11b+) off the viable cells in the spleen of CML, CML mice treated with imatinib (200mg/kg) for 4 weeks and their respective controls. n=5.

B: Representative flow plots for percentage of GFP+ LSCs present in the whole bone marrow of CML-H2b-GFP mice treated with Imatinib (400mg/kg) for 4 weeks. n=2+.

C: Heatmap (left) for the differentially expressed genes and pathway analysis (right) of the upregulated genes from RNA-seq of sensitive and IM-persistent CML cells.

D: Heatmap (left) for the differentially expressed genes and pathway analysis (right) of the upregulated genes obtained from RNA-seq of sorted LSCs from CML mice and CML mice treated with imatinib (200mg/kg) for 4 weeks.

E: Enrichr pathway analysis of the upregulated proteins from RPPA for sensitive and resistant CML cells.

F: Heatmap (top) and KEGG pathway analysis (bottom) after integration of bulk RNA-seq and RPPA for differentially expressed genes which are also activated in K562 cells.

G: String analysis for the upregulated proteins in persistent K562 from Figure 1C. The lines indicate protein interaction while the colors indicate k-means clusters.

The transcriptomic and proteomic data was carried out in triplicates. For scatter plot, two-way ANOVA was used to determine statistical significance with Tukey's multiple comparison. p values < 0.05 were considered statistically significant. *$p < 0.05$, **$p < 0.01$, ***$p < 0.001$, ****$p < 0.0001$; ns = not significant.

SUPPLEMENTAL FIGURE 1

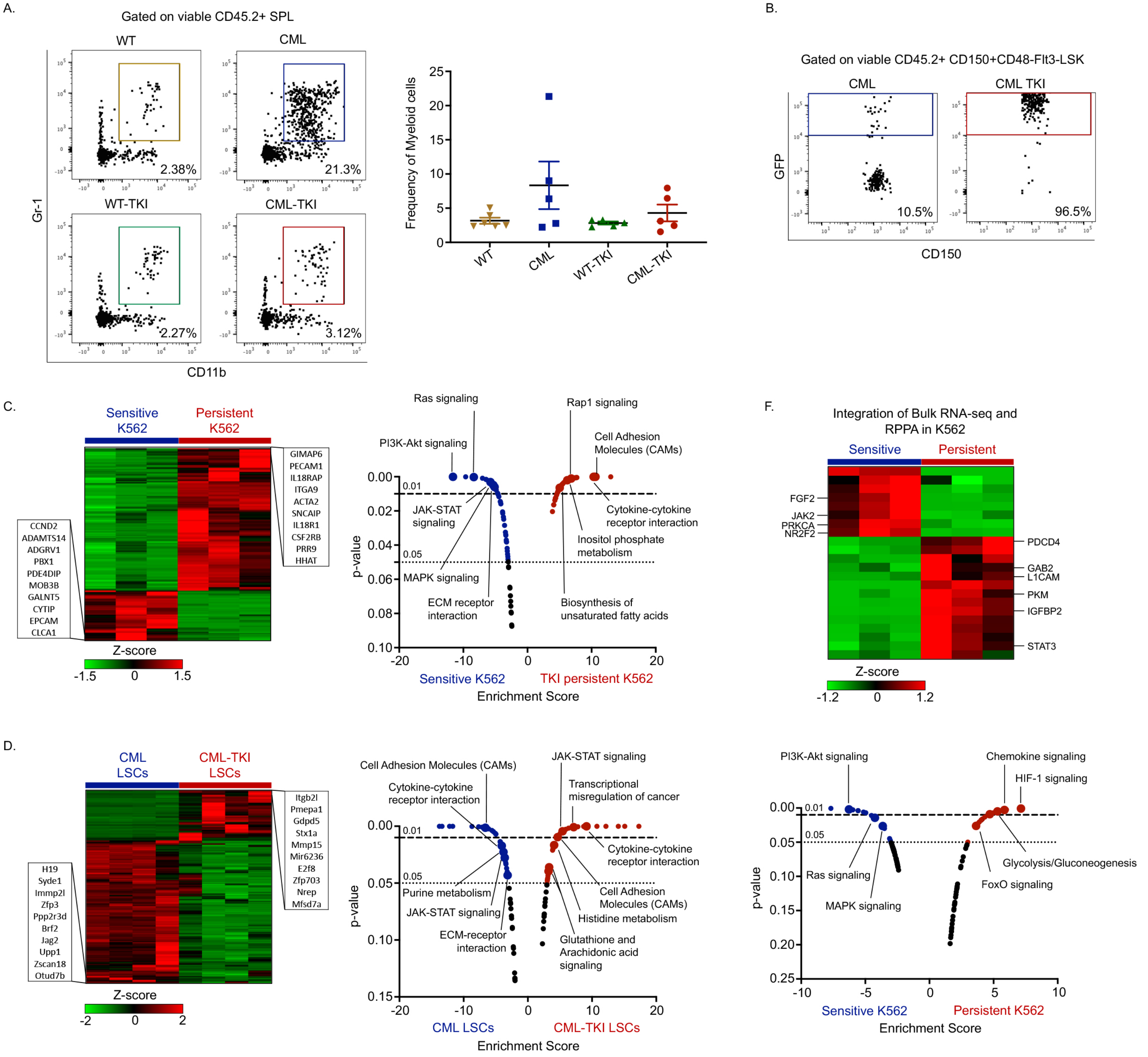

E.

	Enriched Pathway (Panther 2016 human Database)	FDR q-value	Rank
Sensitive K562	EGF receptor signaling pathway	0.0135579	6
	FGF signaling pathway	0.0563876	7
	PDGF signaling pathway	0.0627869	8
Persistent K562	Glycolysis	0.0108899	1
	MAP kinase cascade	0.0160740	3
	Interleukin signaling pathway	0.0633408	13
	Pyruvate metabolism	0.0955132	14

	Enriched Pathway (KEGG 2019 human Database)	FDR q-value	Rank
Sensitive K562	HIF-1 signaling pathway	0.0002627	2
	PI3K-Akt signaling pathway	0.0012289	3
	ErbB signaling pathway	0.0035560	5
	Pathways in cancer	0.0042369	6
Persistent K562	Chronic myeloid leukemia	0.0000071	3
	HIF-1 signaling pathway	0.0000160	8
	FoxO signaling pathway	0.0006697	20
	Phospholipase D signaling pathway	0.0009510	25

G.

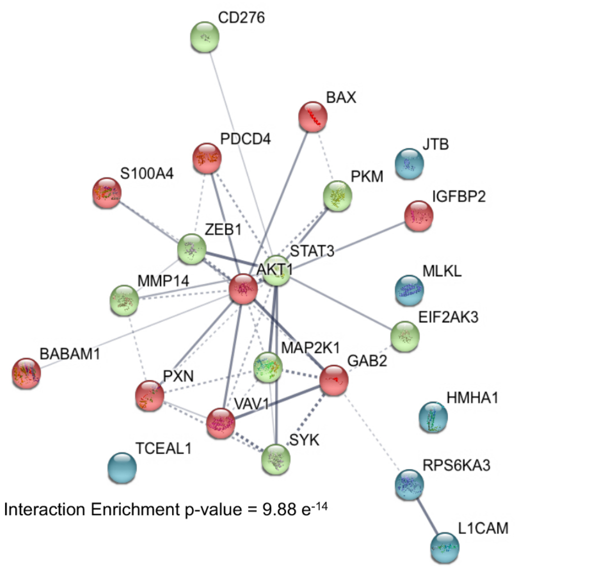

Interaction Enrichment p-value = 9.88 e^{-14}

Figure S2: STAT3 is essential for CML disease progression

A: Western blot of sensitive (Sen) and persistent (Per) K562 lysates for active and total proteins. The bar plot represents density of the persistent protein bands relative to sensitive and normalized to the housekeeping protein, B-actin or GAPDH.

B: 100,000 control and STAT3 Crispr KO cells were treated with gradual increasing doses of imatinib starting 0.2μM and changed to 0.4μM after 2 weeks of exposure to 0.2 μM imatinib. Cells were counted using hemocytometer at the end of 2-week drug treatment.

C: Western blot for single clones of sensitive CML cells to confirm Crispr knock out of STAT3.

D: DNA gel confirming excision of STAT3 post pIC treatment. CD45.2 transplanted CML-$STAT3^{fl/fl}$-MxCre1 and control mice were treated with 100mg/kg pIC for 12 days every other day. DNA was extracted from sorted CD45.2 whole bone marrow and PCR run using primers for excised and full $STAT3^{fl/fl}$ allele.

E: Representative flow plots with frequencies of HSC/LSC for BM stem and progenitor cells in bone marrow (BM) of CML-$STAT3^{fl/fl}$-MxCre1 and control mice. n=3+.

F: Representative flow plots with frequencies of myeloid cells in the whole bone marrow (BM) of CML-$STAT3^{fl/fl}$-MxCre1 and control mice. n=3+.

G: Sensitive and IM-persistent K562 were treated (Rx) with LLL12 (1μM) for 3 days or 6hrs. The lysates were then analyzed by western blot for inhibition of pSTAT3-Y705 and pSTAT3-S727, respectively. Densitometric quantitation was carried out relative to sensitive and normalized to the housekeeping protein, GAPDH.

The data is a representative or a pool of mean ± SD from 2-3 independent experiments. Two-way ANOVA was used to determine statistical significance with Tukey's multiple comparison. p values < 0.05 were considered statistically significant. *$p < 0.05$, **$p < 0.01$, ***$p < 0.001$, ****$p < 0.0001$; ns = not significant.

SUPPLEMENTAL FIGURE 2

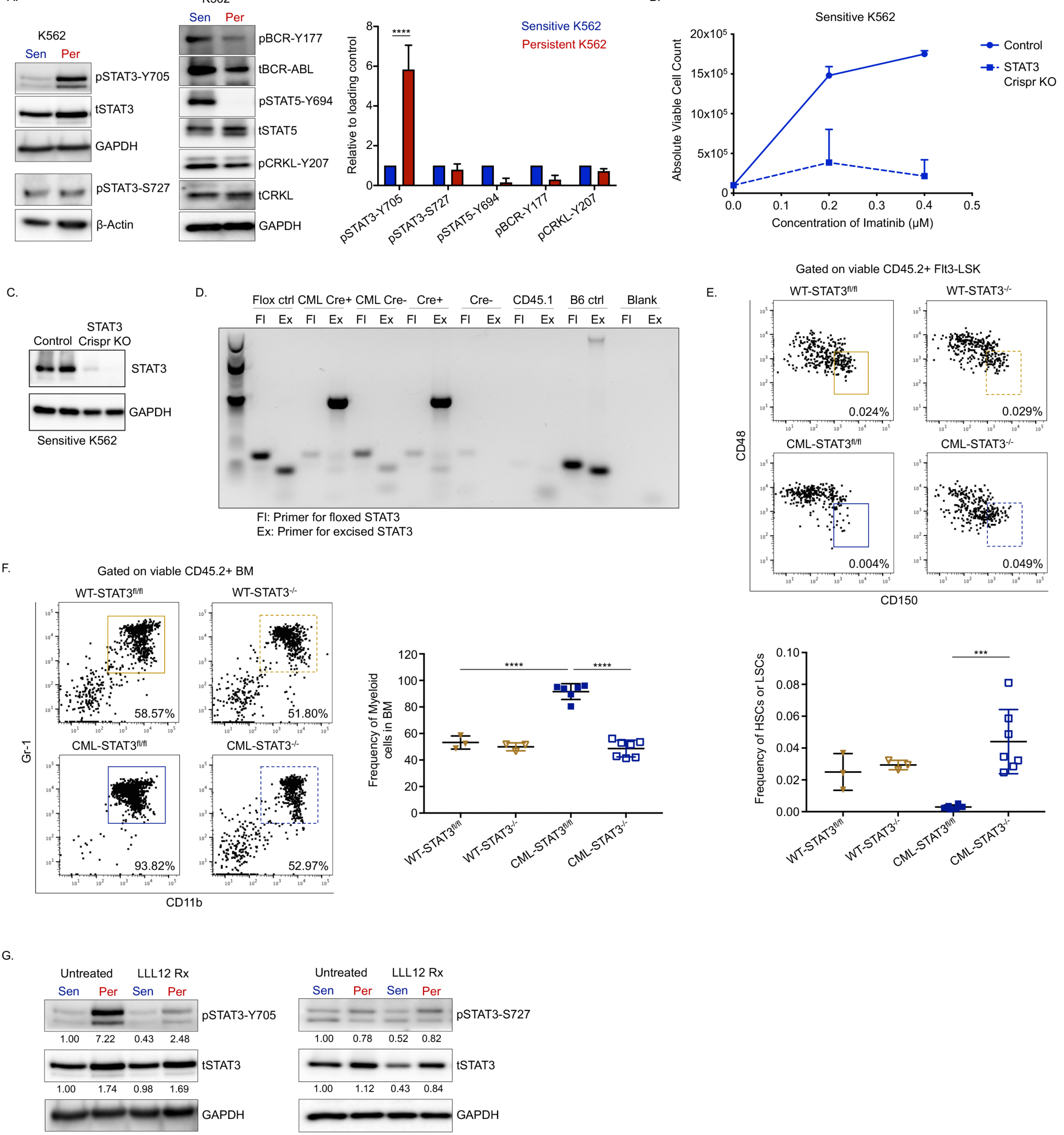

Figure S3: STAT3 localizes to unique sites in the TKI persistent CML stem and progenitor cells

A: Venn diagram for the number peaks detected for pSTAT3-Y705 localizing to the genome from WT, CML and IM-persistent CML LSK.

B: Motif analysis for pSTAT3-Y705 localizing site in CML and IM-persistent CML LSK compared to the known consensus site obtained from GAS and Jasper-GAS.

The transcriptomic data was carried out in triplicates.

SUPPLEMENTAL FIGURE 3

A.

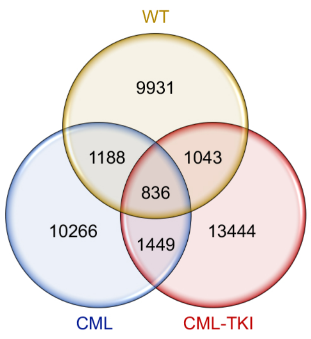

B.

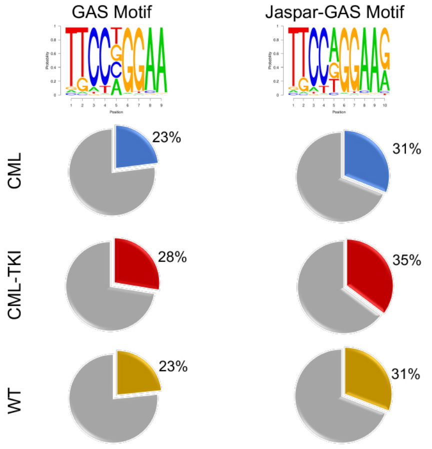

Figure S4: TKI persistent K562 have altered mitochondrial metabolism

A: Mitochondrial DNA (mtDNA) copy number relative to nuclear DNA for sensitive and IM-persistent K562.

B: Transmission Electron Microscopy images of sensitive and IM-persistent K562. Mitochondria were counted manually while the mitochondrial area was calculated using ImageJ analysis. n=10.

C: Western blot for ETC complexes using sensitive and IM-persistent K562 cell lysate. The bar plot represents density of the resistant protein bands relative to sensitive and normalized to the housekeeping protein, B-actin.

D: Western blot of whole cell lysate, cytoplasmic fraction and mitochondrial fraction for VDAC2 and B-actin to confirm the purity of mitochondrial extract. Mitochondria were isolated from 20 million sensitive and IM-persistent K562. Post mitochondrial purity check, lysates were blotted for pSTAT3-S727, acSTAT3-K685 and tSTAT3, with calrecticulin used as a housekeeping protein.

E: Heatmap for the top 50 differentially expressed metabolites between WT HSCs and CML LSCs; and LSCs obtained from CML mice and CML mice treated with IM. Sum normalized and auto-scaled values from Metaboanalyst were used in the heatmap.

F: PLS-DA plot comparing WT HSCs, CML LSCs, IM-persistent CML LSCs.

G: PLS-DA plot combining steady-state metabolites in sensitive and IM-persistent K562 versus murine CML LSCs and IM-persistent CML LSCs.

H: OCR from MST (left) and ECAR from GRA (right) of sensitive and IM-persistent measured by seahorse after treating the cells with LLL12 (1μM) for 3 days.

The data is a representative or a pool of mean ± SD from 2-3 independent experiments. Unpaired student t-test or Two-way ANOVA was used to determine statistical significance with Tukey's multiple comparison. p values < 0.05 were considered statistically significant. *$p < 0.05$, **$p < 0.01$, ***$p < 0.001$, ****$p < 0.0001$; ns = not significant.

SUPPLEMENTAL FIGURE 4

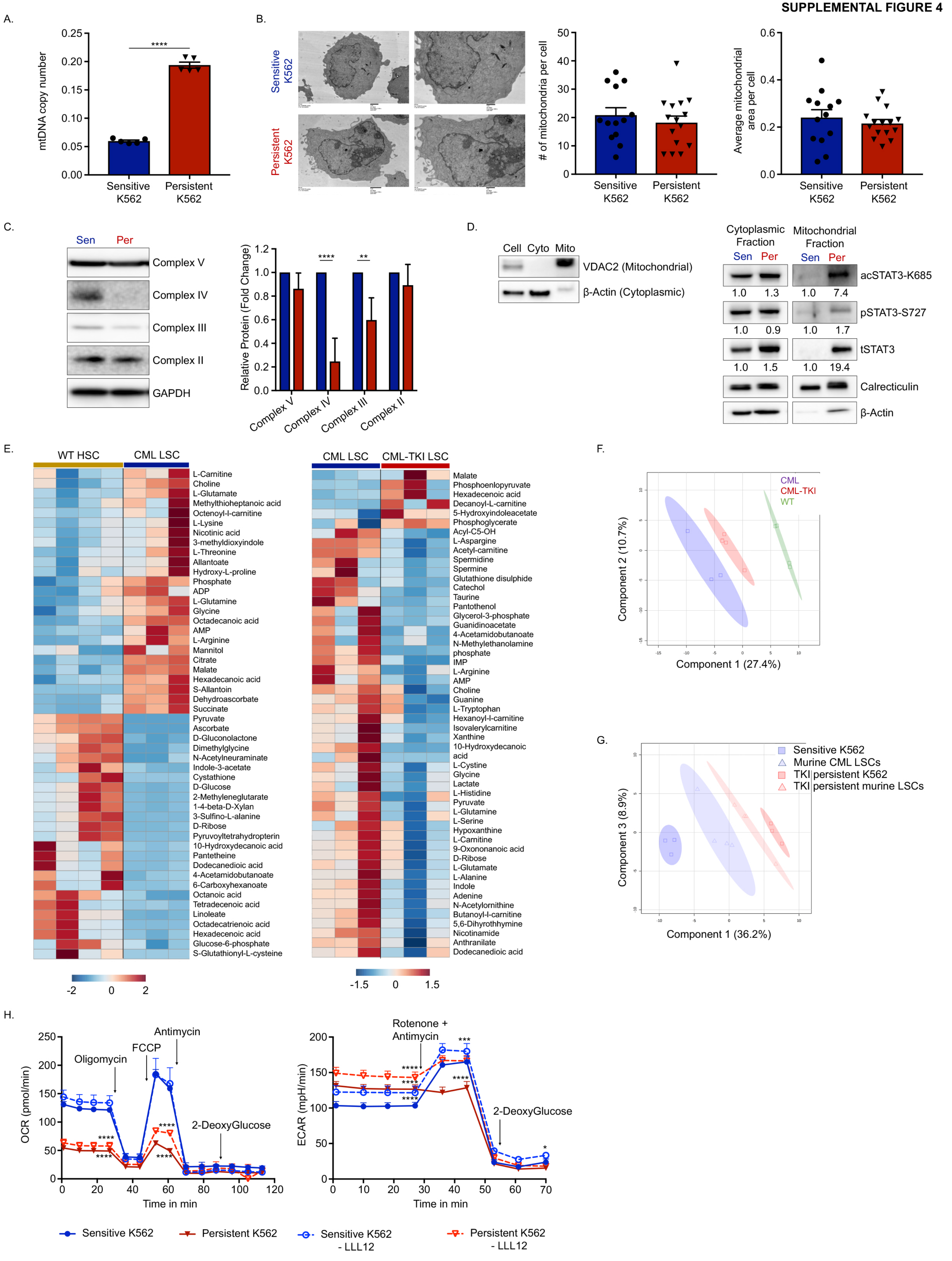

Figure S5: TKI persistent K562 have active reductive carboxylation

A: Unit area plots for metabolites labeled post 3hr incubation of 200,000 sensitive and IM-persistent K562 with isotopically labeled $^{13}C_{1,2,3}$-glucose. The metabolites were measured with UHPLC-MS.

B: Ratio of a-ketoglutarate and citrate in the sensitive and IM-persistent K562 obtained from stable metabolic profiles in Fig S4H.

C and D: Unit area plots for metabolites labeled in oxidative (C) and reductive (D) glutamine metabolism post 12hr incubation of 200,000 sensitive and IM-persistent K562 with isotopically labeled $^{13}C_5{}^{15}N_2$-glutamine. The metabolites were measured with UHPLC-MS.

E: Unit area plots for metabolites labeled in fatty acid oxidation post 72hr incubation of 200,000 sensitive and IM-persistent K562 with isotopically labeled U-$^{13}C_{16}$-sodium palmitate. The metabolites were measured with UHPLC-MS.

The data is representative or a pool of mean ± SD from 2 independent experiments. Unpaired student t-test or Two-way ANOVA was used to determine statistical significance with Tukey's multiple comparison. p values < 0.05 were considered statistically significant. *p < 0.05, **p <0.01, ***p < 0.001, ****p < 0.0001; ns = not significant.

SUPPLEMENTAL FIGURE 5

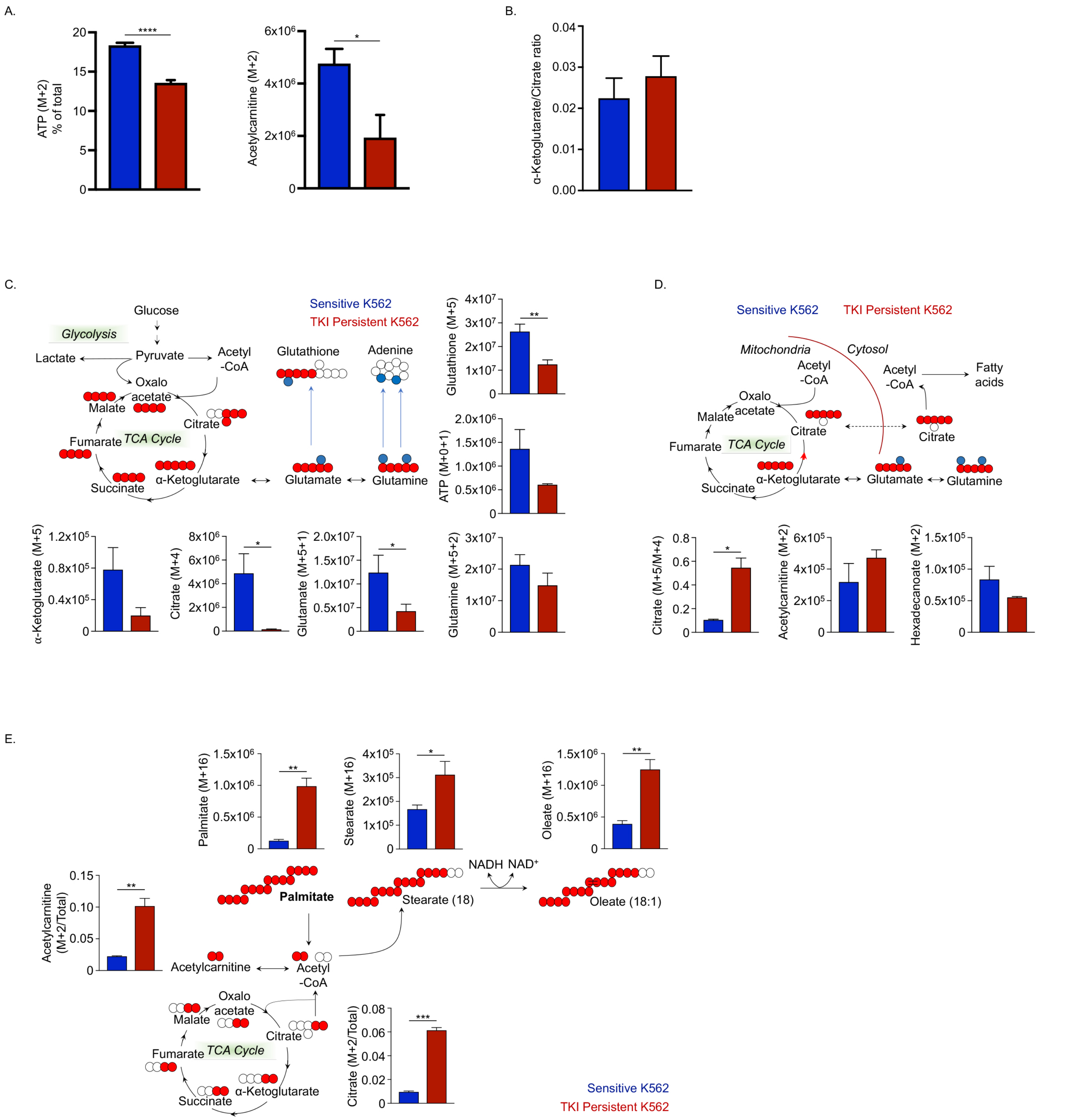

Figure S6: TKI persistent LSCs have differential expression of glycolytic genes

A: Heatmap depicting the expression of hallmark glycolysis/gluconeogenesis genes in murine CML LSCs and CML-IM LSCs.

B: AnnexinV apoptosis assay for sensitive and IM-persistent K562 treated with increasing dose of 2-Dexoyglucose (2-DG) from 0 to 50mM and analyzed by flow cytometry after 48hrs.

C: Sensitive and IM-persistent K562 were treated with compound 3K (0.5μM) for 18hrs. The lysates were then analyzed by western blot for inhibition of PKM2. Densitometric quantitation was carried out relative to sensitive and normalized to the housekeeping protein, GAPDH.

D: Dose curve of compound 3K for sensitive and IM-persistent K562 untreated or treated for 3 days. The cells were stained with AnnexinV for apoptosis and analyzed by flow cytometry.

E: Sensitive and IM-persistent K562 were treated with Shikonin (0.5μM) for 6hrs. The lysates were then analyzed by western blot for inhibition of PKM2. Densitometric quantitation was carried out relative to sensitive and normalized to the housekeeping protein, GAPDH.

F: Dose curve of Shikonin for sensitive and IM-persistent K562 untreated or treated for 3 days. The cells were stained with AnnexinV for apoptosis and analyzed by flow cytometry.

G: Percentage of apoptotic LSK cells treated with imatinib (1μM) or shikonin (0.5μM) alone or in combination for 48hrs in hypoxia. 10,000 sorted LSK from control, CML and imatinib (400mg/kg) treated CML mice were used for the experiment, stained with AnnexinV and analyzed by flow cytometry. n=3.

H: Viable cell counts of human CD34+ BM cells treated with imatinib (1μM) or shikonin (0.5μM) alone or in combination for 48hrs in hypoxia. 5,000-20,000 sorted CD34+ cells from 2 healthy individuals, 5 CML patients at diagnosis and 1 IM treated disease persistent patient were used for the experiment, stained with AnnexinV and analyzed by flow cytometry. Data is represented as counts normalized untreated.

I: Percentage of apoptotic human CD34+ BM cells treated with imatinib (1μM) or shikonin (0.5μM) alone or in combination for 48hrs in hypoxia. 5,000-20,000 sorted CD34+ cells from 5 CML patients at diagnosis were used for the experiment, stained with AnnexinV and analyzed by flow cytometry.

The data is representative or pool of mean ± SD from 2-3 independent experiments. Unpaired student t-test or Two-way ANOVA was used to determine statistical significance with Tukey's multiple comparison. p values < 0.05 were considered statistically significant. *p < 0.05, **p <0.01, ***p < 0.001, ****p < 0.0001; ns = not significant.

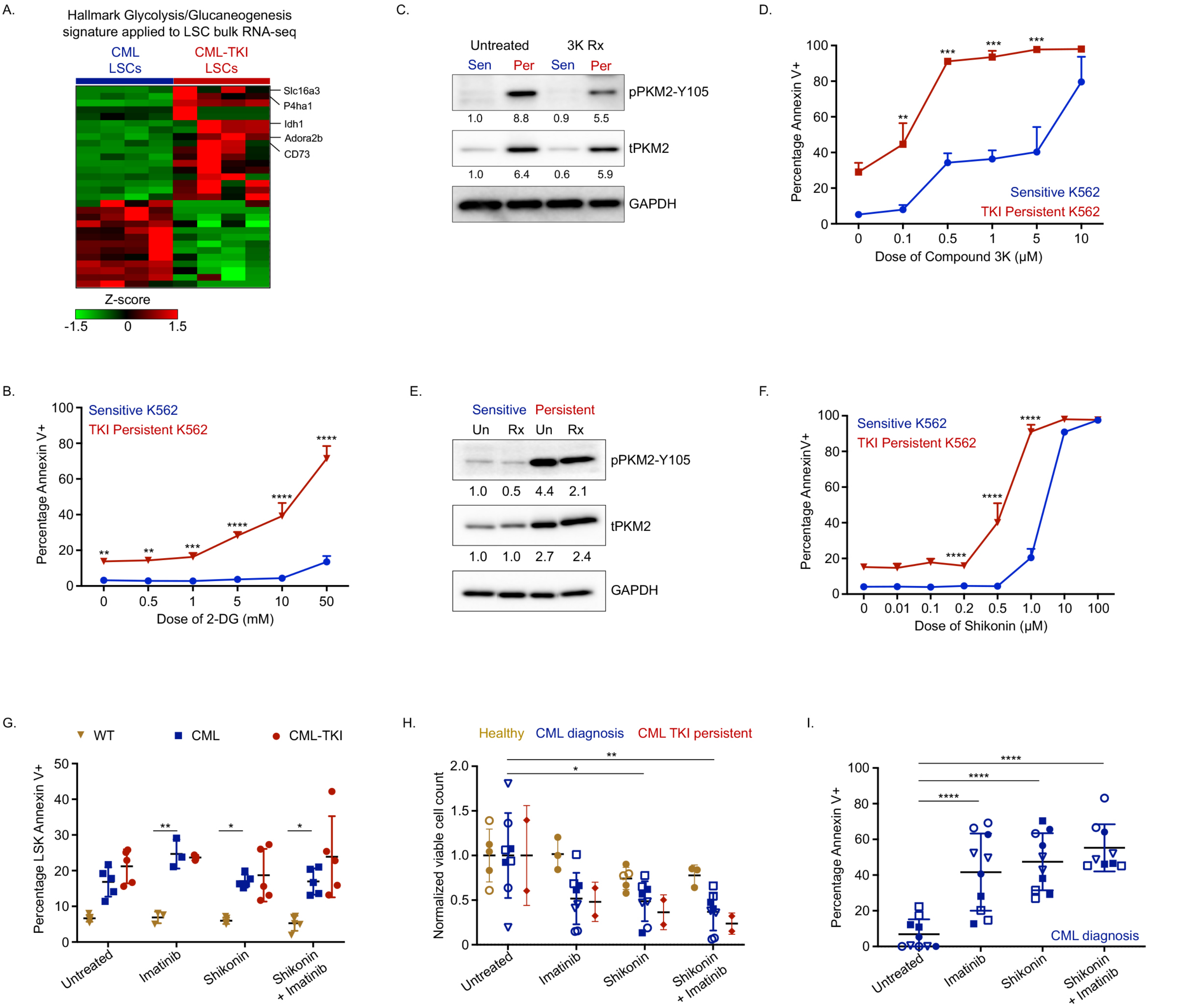
A.
Hallmark Glycolysis/Glucaneogenesis
signature applied to LSC bulk RNA-seq
CML
LSCs
CML-TKI
LSCs
Slc16a3
P4ha1
Idh1
Adora2b
CD73
Z-score
-1.5
0
1.5
C.
Untreated
3K Rx
Sen
Per
Sen
Per
pPKM2-Y105
1.0
8.8
0.9
5.5
tPKM2
1.0
6.4
0.6
5.9
GAPDH
D.
Percentage Annexin V+
Sensitive K562
TKI Persistent K562
Dose of Compound 3K (μM)
B.
Sensitive K562
TKI Persistent K562
Percentage Annexin V+
Dose of 2-DG (mM)
E.
Sensitive
Persistent
Un
Rx
Un
Rx
pPKM2-Y105
1.0
0.5
4.4
2.1
tPKM2
1.0
1.0
2.7
2.4
GAPDH
F.
Sensitive K562
TKI Persistent K562
Percentage Annexin V+
Dose of Shikonin (μM)
G.
WT
CML
CML-TKI
Percentage LSK Annexin V+
Untreated
Imatinib
Shikonin
Shikonin + Imatinib
H.
Healthy
CML diagnosis
CML TKI persistent
Normalized viable cell count
Untreated
Imatinib
Shikonin
Shikonin + Imatinib
I.
Percentage Annexin V+
CML diagnosis
Untreated
Imatinib
Shikonin
Shikonin + Imatinib

REFERENCES

1. Eiring AM, Page BD, Kraft IL, Mason CC, Vellore NA, Resetca D, et al. Combined STAT3 and BCR-ABL1 inhibition induces synthetic lethality in therapy-resistant chronic myeloid leukemia. Leukemia. 2015;29(3):586-97.

2. Chu S, McDonald T, Lin A, Chakraborty S, Huang Q, Snyder DS, et al. Persistence of leukemia stem cells in chronic myelogenous leukemia patients in prolonged remission with imatinib treatment. Blood. 2011;118(20):5565-72.

3. Pietras EM, Reynaud D, Kang YA, Carlin D, Calero-Nieto FJ, Leavitt AD, et al. Functionally Distinct Subsets of Lineage-Biased Multipotent Progenitors Control Blood Production in Normal and Regenerative Conditions. Cell stem cell. 2015;17(1):35-46.

4. Szilvassy SJ, Humphries RK, Lansdorp PM, Eaves AC, Eaves CJ. Quantitative assay for totipotent reconstituting hematopoietic stem cells by a competitive repopulation strategy. Proceedings of the National Academy of Sciences of the United States of America. 1990;87(22):8736-40.

5. Welner RS, Amabile G, Bararia D, Czibere A, Yang H, Zhang H, et al. Treatment of chronic myelogenous leukemia by blocking cytokine alterations found in normal stem and progenitor cells. Cancer cell. 2015;27(5):671-81.

6. Cappella P, Gasparri F, Pulici M, Moll J. A novel method based on click chemistry, which overcomes limitations of cell cycle analysis by classical determination of BrdU incorporation, allowing multiplex antibody staining. Cytometry Part A : the journal of the International Society for Analytical Cytology. 2008;73(7):626-36.

7. Bararia D, Kwok HS, Welner RS, Numata A, Sarosi MB, Yang H, et al. Acetylation of C/EBPalpha inhibits its granulopoietic function. Nature communications. 2016;7:10968.

8. Iadevaia S, Lu Y, Morales FC, Mills GB, Ram PT. Identification of optimal drug combinations targeting cellular networks: integrating phospho-proteomics and computational network analysis. Cancer Res. 2010;70(17):6704-14.

9. Cheng KW, Lu Y, Mills GB. Assay of Rab25 function in ovarian and breast cancers. Methods Enzymol. 2005;403:202-15.

10. Tibes R, Qiu Y, Lu Y, Hennessy B, Andreeff M, Mills GB, et al. Reverse phase protein array: validation of a novel proteomic technology and utility for analysis of primary leukemia specimens and hematopoietic stem cells. Molecular cancer therapeutics. 2006;5(10):2512-21.

11. Ran FA, Hsu PD, Wright J, Agarwala V, Scott DA, Zhang F. Genome engineering using the CRISPR-Cas9 system. Nat Protoc. 2013;8(11):2281-308.

12. Hill BG, Benavides GA, Lancaster JR, Jr., Ballinger S, Dell'Italia L, Jianhua Z, et al. Integration of cellular bioenergetics with mitochondrial quality control and autophagy. Biol Chem. 2012;393(12):1485-512.

13. Redmann M, Benavides GA, Wani WY, Berryhill TF, Ouyang X, Johnson MS, et al. Methods for assessing mitochondrial quality control mechanisms and cellular consequences in cell culture. Redox Biol. 2018;17:59-69.
